# Iatrogenic QT Abnormalities and Fatal Arrhythmias: Mechanisms and Clinical Significance

**DOI:** 10.2174/157340309788970397

**Published:** 2009-08

**Authors:** Luigi X Cubeddu

**Affiliations:** Nova Southeastern University, HPD, Cardiovascular and Metabolic Research Division, Fort Lauderdale, FL, USA

**Keywords:** Long QT, drug-induced, HERG potassium channels, torsades de pointe.

## Abstract

Severe and occasionally fatal arrhythmias, commonly presenting as Torsade de Pointes [TdP] have been reported with Class III-antiarrhythmics, but also with non-antiarrhythmic drugs. Most cases result from an action on K^+^ channels encoded by the HERG gene responsible for the IKr repolarizing current, leading to a long QT and repolarization abnormalities. The hydrophobic central cavity of the HERG-K+ channels, allows a large number of structurally unrelated drugs to bind and cause direct channel inhibition. Some examples are dofetilide, quinidine, sotalol, erythromycin, grepafloxacin, cisapride, dolasetron, thioridazine, haloperidol, droperidol and pimozide. Other drugs achieve channel inhibition indirectly by impairing channel traffic from the endoplasmic reticulum to the cell membrane, decreasing channel membrane density (pentamidine, geldalamicin, arsenic trioxide, digoxin, and probucol). Whereas, ketoconazole, fluoxetine and norfluoxetine induce both direct channel inhibition and impaired channel trafficking. Congenital long QT syndrome, subclinical ion-channel mutations, subjects and relatives of subjects with previous history of drug-induced long QT or TdP, dual drug effects on cardiac repolarization [long QT plus increased QT dispersion], increased transmural dispersion of repolarization and T wave abnormalities, use of high doses, metabolism inhibitors and/or combinations of QT prolonging drugs, hypokalemia, structural cardiac disease, sympathomimetics, bradycardia, women and older age, have been shown to increase the risk for developing drug-induced TdP. Because most of these reactions are preventable, careful evaluation of risk factors and increased knowledge of drugs use associated with repolarization abnormalities is strongly recommended. Future genetic testing and development of practical and simple provocation tests are in route to prevent iatrogenic TdP.

## INTRODUCTION

The potentially dangerous proarrhythmic effects of drugs have been recognized since the days when digitalis and quinidine toxicities were described [1]. Subsequently, with widespread use of newer antiarrhythmic drugs, in particular agents with class III activity, many cases of drug-induced arrhythmia were documented [1]. However, not until recently we have become aware that many non-antiarrhythmic drugs have proarrhythmic activities and in fact have been associated with severe and occasionally fatal arrhythmias, commonly presenting as Torsade de Pointes (TdP) [1-6].

Several agents of distinct pharmacological classes have been removed from the market or their use limited because of deaths related to ventricular arrhythmias. Among these agents are antibiotics (Grepafloxazin - Raxar®), prokinetics (Cisapride - Propulsid®), antihistaminics (Terfenadine - Seldane®; Astemizole - Hismanal®), and antipsychotics (Sertindole - Serlect^®^) [1, 2]. Cisapride, for example, is available only through a limited-access program and its use is indicated for patients who have failed other therapies, have a normal ECG and in consultation with a specialist. Warnings have also been issued for droperidol because of its propensity to induce QT prolongation and fatal arrhythmia [7].

TdP was first described by Dessertenne [8, 9] as a “polymorphic ventricular tachycardia characterized by an electrocardiographic pattern of continuously changing morphology of QRS complexes that seem to twist around the imaginary baseline”. TdP may manifest as palpitations, syncope, seizure-like activity, cardiac arrest requiring resuscitation and defibrillation, and/or as sudden cardiac death. It is thus apparent, that although TdP may present as a short-lived and self-limited arrhythmia, it may also lead to fatal events [1-5].

Information about the occurrence of TdP in the general population is unknown. Although TdP is reported to occur in 4 out of 100,000 individuals per year, a higher rate would be expected in predisposed patient groups [10].

TdP is commonly associated with acquired or hereditary forms of prolonged QT interval (long QT syndrome, LQTS). TdP often starts after the T wave of a markedly prolonged QT interval that followed a cycle that had been prolonged, usually by a post-ectopic pause. Among the conditions, drug-induced long QT is by far the most common, and hence, preventable. Recent findings suggest that many of the acquired forms of TdP develop in individuals who harbor subclinical congenital abnormalities. In addition to drugs, other predisposing factors for prolonging the QT interval and inducing TdP are electrolyte abnormalities and structural cardiac disorders. Coexistence of several of the predisposing factors in a patient increases the likelihood of developing long QT and TdP. Although the duration of the QT interval was considered as the best clinical marker for the risk of TdP [1], increased variability of QT duration (repolarization inhomogeneity) and T wave abnormalities have also been associated with increased for TdP [5, 11, 12] (Table **1**).

## MECHANISMS OF LONG QT, REPOLARIZATION ABNORMALITIES AND TDP

The width of the ventricular action potential determines the duration of the QT interval on the ECG. In contrast with QRS interval, the duration of the QT interval varies from beat to beat, shows circadian variation and is affected by changes in the heart rate and autonomic tone. The faster the heart rate the shorter the QT interval, consequently, a rate corrected QT [QTc] is commonly employed [13, 14]. QTc values greater than 450 msec for men and 470 msec for women are usually considered as abnormally prolonged intervals [15]. Recent findings suggest that QT interval variation in the general population is largely heritable [16].

Ventricular repolarization is mainly due to outward K^+^ currents. Two main delayed rectifying currents operate to achieve repolarization, a rapid [IKr] and a slow [IKs] current. When these currents are reduced, repolarization is prolonged, the ventricular action potentials broaden and the duration of the QT interval increases. When the period of repolarization is prolonged, depolarizing currents may ensue, which when repeated and of sufficient magnitude, may trigger a ventricular arrhythmia. Both early and late depolarizing currents have been described. TdP is classically associated with early-afterdepolarizations, which are depolarizing currents occurring early during the period of repolarization [17-19], due mostly to inward calcium currents. During delayed repolarization, inactive L-type calcium channels may become active, leading to inward depolarizing calcium currents [19]. Therefore, conditions or treatments that increase the number of L-type calcium channels in cardiac fiber membranes, such as sympathetic stimulation or administration of beta-receptor agonists, may facilitate the induction of TdP in the presence of delayed repolarization. Clinically, the early depolarizations correlate well with the presence of T wave humps, T-U waves or bifid T waves that often precede the development of TdP [18].

Interference with the IKr current leading to prolonged repolarization and long QT is the most common mechanism of congenital and drug-induced triggered arrhythmia [20-22]. The IKr repolarizing current is conducted through K^+^ channels that are coded by the human human-ether-a-go-go-related gene (HERG). In addition to prolonged repolarization [i.e., long QT], the interlead variation of QT duration in a surface electrocardiogram also known as QT dispersion or variability predicts risk of TdP [12, 23]. QT dispersion is indicative of spatial heterogeneity of repolarization times in the ventricular muscle. Differences in the time course of repolarization of the cell layers that make up the ventricular myocardium give rise to transmural voltage gradients and a dispersion of repolarization. QT dispersion is commonly calculated from the difference between the maximum and the minimum QTc in any thoracic lead. Values greater than 80 msec are considered abnormally prolonged [24]. In the presence of a prolonged QT interval, repolarization inhomogeneity increases the risk of TdP [5]. Bradycardia is associated with increased risk for developing TdP due to a combination of prolonged QT and increased transmural dispersion of repolarization. Further, agents that prolong the QT interval but fail to increase the transmural dispersion of repolarization may not induce TdP [25]. Consequently, EKG assessments of increased risk of TdP should include: QT interval duration, beat-to-beat QT variability (QT dispersion), interval from peak to the end of the T wave, presence T-wave alternans [change in amplitude or polarity of T wave on alternating beats] and “T-U waves” [26, 27] (Table **1**).

The significance of repolarization abnormalities on outcomes was recently assessed. Long QT (>440msec) associated with high β-natriuretic peptide levels, were found to be an independent predictor of mortality in advanced CHF and in heart transplant patients [28, 29]. Additionally, QT duration and dispersion abnormalities were found to be significant predictors of cardiac mortality in the general population as well as in diabetics [30, 31] (Table **1**).

## GENETIC BASIS FOR IATROGENIC-LONG QT AND TDP

In the absence of structural disease (ischemic heart disease, congestive heart failure, cardiomyopathies), marked bradycardia, electrolyte abnormalities such as low potassium and low magnesium, subarachnoid hemorrhage, human immunodeficiency virus disease [32], or drug therapy, the presence of an abnormally prolonged QTc interval is suggestive of congenital long QT [33-35]. Genetic predisposition and acquired risk actors interact in an individual to produce an increased risk of TdP arrhythmia. The concept of repolarization reserve has been employed to account for individual differences in the susceptibility to develop TdP with drugs or conditions known to affect K^+^ currents.

Mutations in the genes coding for transmembrane ion-channel proteins cause most cases of congenital long QT syndrome [36]. Seven different genes and over 400 mutations have been identified so far [36]. Mutations in potassium channel genes (KCNQ1, KCNH2, KCNE1, KCNE2, KCNJ2), as well as in sodium (SCN5A) and calcium channel genes (CACNA1C) have been described [36-38]. However, 90% of all cases have defects in genes coding for ion channels involved in repolarizing K^+^ currents: the IKs ion channels (LQTS1, chromosome 11) of this ion channels (LQTS2, chromosome 7). HERG alpha-subunits and MiRP1 (MinK-related peptide beta-subunits-1) constitute the native human IKr. Mutations of HERG and MiRP1 decrease the repolarizing current, delay ventricular repolarization and prolong the QT interval [21, 37, 38]. Few patients have defects in genes coding for sodium channels (LQTS3, chromosome 5). Patients with the congenital forms of the LQTS may present with frequent episodes of arrhythmias and syncope associated with TdP [1]. In some, the arrhythmias are triggered by exercise, whereas in others arrhythmias are independent of physical activity presenting at night and at rest. Arrhythmias triggered by auditory stimuli, low serum potassium and clarithromycin have also been reported. In the near future, the management of LQTS may be greatly facilitated by recognition of the type of mutation present in an individual. For example, LQTS subjects with a MiRP1 mutation in the potassium channel should avoid clarithromycin and macrolide antibiotics [39].

In addition to mutations known to affect the function of the ion channels (loss of function mutations), mutations leading to a reduced number of functional channels or in the rate of incorporation of channels into the cell membrane may also lead to severe and fatal arrhythmias [40]. Assembled channels are normally transferred from the endoplasmic reticulum into the cell membrane. Channel mis-processing and/or abnormal channel trafficking may affect ion currents [40]. For example, subjects with LQTS3 have a mutation on the SCN5A cardiac sodium channel. Such a mutation delays the incorporation of the sodium channels into the cell membrane, leading to late depolarizing currents, which may trigger an arrhythmia. Similarly, subjects with LQTS2 harboring HERG N470D and HERG R752W mutations have impaired trafficking of potassium channels from the endoplasmic reticulum to the cell surface, reducing the density of channels in the cell surface, and thus presenting as a long QT syndrome [41-44]. Because the mutated channels are retained in the endoplasmic reticulum, strategies are being developed to restore normal trafficking. Several chemical chaperones and some HERG- K^+^ channel blockers, such as astemizole and cisapride, have been shown to rescue HERG K^+^ channels under *in vitro* conditions [43]. Unfortunately, the clinical usefulness of aztemizole and cisapride to correct defective channel trafficking is limited by their stronger potency as channel blockers than as channel trafficking stabilizers. Selective HERG K^+^ channel rescue agents lacking ion channel blocking activity are being currently developed [45].

## SERUM POTASSIUM, LONG QT AND TDP

Low levels of serum K^+^ are associated with increased risk for TdP (Table **1**). Unlike most K^+^ currents, the magnitude of the IKr current is reduced by low extracellular K^+^ concentrations, further prolonging repolarization [46]. This probably explains the marked QT prolongation and the induction of TdP observed in patients receiving an IKr antagonist in the presence of low serum K^+^ [47]. As for drugs that inhibit the IKr currents, the arrhythmogenic effect of low serum K^+^ is enhanced in the presence of bradycardia [35, 46]. Reductions in serum K^+^ are also known to increase the incidence of arrhythmia in subjects with a LQTS. Choi and colleagues [48] studied the effects of a K^+^ infusion in healthy volunteers and in CHF patients during a challenge with quinidine. K^+^ administration (0.5 mEq/kg, maximum 40 mEq, in 0.9% saline in one-hour infusion) reduced the duration and dispersion of the QTc interval, and reversed the morphological QT abnormalities, including U waves and bifid T waves in both groups of subjects. These beneficial ECG changes were observed with increases in serum K^+^ of 0.4 to 0.7 mEq/lt, and with baseline serum K^+^ within normal levels. Increasing the serum K^+^ from low normal (4.1 mEq/lt) to high normal (4.8 mEq/lt) reduced QTc duration by approximately 100 msec [48]. Increases in serum K^+^ to levels above 4.0 mEq/lt have also been reported to correct the ECG abnormalities in patients with congenital forms of the long QT syndrome. ECG abnormalities of subjects with LQTS2 due to mutations in the HERG gene were corrected by K^+^ treatment [49]. It is proposed that the conventional lower limit for serum K^+^ should be raised in patients with LQTS, either congenital or acquired, as well as in subjects scheduled to receive treatment with drugs known to prolong the QT interval.

## MECHANISMS OF DRUG-INDUCED REPOLARIZATION ABNORMALITIES AND INCREASED RISK FOR TDP

As with congenital forms of long QT syndrome, most cases of drug-induced long QT and TdP result from an action of the drugs on the ion channel proteins encoded by the HERG gene that is responsible for the IKr repolarizing current. Drug-induced QT prolongation is most commonly achieved by direct channel blockade. Interestingly, contrary to most drug-receptor interactions, binding to the HERG K^+^ channels seems to be quite unspecific. A large number of structurally unrelated drugs exert direct blockade of the HERG K^+^ channels. Such unspecific drug-channel interaction seems to result from the large number of aromatic residues present in the K^+^ channel compared with other ion-channels [44]. It has been proposed that the hydrophobic central cavity of the HERG K^+^ channels may stabilize the binding of drugs to the channel protein leading to IKr inhibition [44].

In addition to drug-induced direct HERG K^+^ channel blockade, some agents (Table **2**) induce repolarization abnormalities by inhibiting channel trafficking, reducing the incorporation of K^+^ channels into the cell membrane [20-22, 34]. Drugs such as ketoconazole, fluoxetine, and norfluoxetine induce QT-prolongation via a dual mechanism, i.e., direct channel blockade and inhibition of cellular channel trafficking (Table **2**). It is expected that the list of drugs with effects on HERG K^+^ channel trafficking will increase, as more agents are tested for such an action.

Although many drugs inhibit the IKr, fortunately only very few patients develop TdP when treated with QT-prolonging drugs [5]. The low incidence and often-unpredictable emergence of TdP may result from individual differences in pharmacokinetics and/or pharmacogenomics. In this regard, it has been proposed that subjects susceptible to drug-induced TdP (i.e, low repolarization reserve) may have mutations in the HERG K^+^ channel, which would become apparent when exposed to a QT-prolonging drug [5, 50]. Up to 20% of people developing drug-induced TdP were found to have a low repolarization reserve due to subclinical ion-channel mutations [51]. In addition to genetic factors, drugs may exert multiple actions on cardiac repolarization. The increased risk for developing TdP with high doses of sotalol will result from a dual drug effect on cardiac repolarization, i.e., prolonged repolarization and increased repolarization heterogeneity. Conversely, concomitant drug actions on ion channels (i.e., calcium channel blockade) and receptors (beta receptor antagonism) may override the risk of TdP associated with IKr inhibition. Amiodarone and verapamil exemplify this situation. These findings also indicate that a normal QT at baseline does not preclude excessive QT prolongation and even development of TdP when exposed to a QT-prolonging drug [52]. Importantly, a previous history of drug-induced TdP should preclude the use of a QT prolonging drug.

## DRUG CLASSES ASSOCIATED WITH LONG QT AND TDP

Antiarrhythmic drugs with class III activity (IKr blockade) are expected to increased risk for developing TdP [34, 53] (Table **3**). These agents act as antiarrhythmics by inhibiting outward K^+^ current, delaying repolarization, prolonging the QT interval and increasing refractoriness. Other antiarrhythmics with IKr blocking properties, such as class Ia drugs, bepridil and sotalol also prolong the QT interval. Quinidine, dofetilide and ibutilide, are all associated with TdP and are potent blockers of the IKr channel; thus, for these three agents there is a strong association between these two variables [46]. Quinidine, not only prolongs the duration of QT interval but also increases the transmural dispersion of repolarization at slow rather than at faster heart rates. Verapamil, on the other hand, is a drug not associated with TdP, but it also blocks IKr currents. However, verapamil blocks L-type calcium channels and additionally decreases transmural dispersion of repolarization; the combi-nation of both factors may account for the low incidence of TdP associated with its use [54].

An interesting example of class III drugs is sotalol; which has been associated with TdP. Sotalol is composed on a racemic mixture of D- and L-sotalol. D-sotalol inhibits the IKr and prolongs the QT interval. L-sotalol, on the other hand, is a beta-blocker. The racemic mixture has been reported to be less arrhythmogenic than the D-sotalol. The SWORD trial reported an increased mortality when D-sotalol was employed in patients with left ventricular dysfunction after recent or remote myocardial infarction [55]. The lesser effect of the racemic mixture is most likely due to the beta-blocking properties of L-sotalol. Sotalol-induced recurrent TdP has been observed in patients with end-stage renal disease, which may result from very high plasma levels of sotalol, since the drug is not adequately eliminated by renal dialysis [56]. At high concentrations, sotalol not only prolongs the QT interval but in addition it increases beat-to-beat QT interval variability (repolarization heterogeneity), increasing the likelihood of developing TdP [57].

Amiodarone exerts inhibitory effects on the IKr and the IKs currents, prolonging repolarization and the QT interval [58]. Despite the bradycardia and the large QT prolongation associated with chronic amiodarone therapy, TdP and other drug-induced tachyarryhthmias are unusual. Amiodarone reduces both heterogeneity of repolarization across cell type within myocardium (epi, endo and mid-myocardial cells) and QT variability [57]. These effects may account for the lower than expected incidence of TdP observed with the drug. Additionally, amiodarone exerts alpha and beta blocking properties and calcium channel blocking activity, as well as multiple antiarrhythmic activities, which may also lower the risk of TdP [46, 59]. As with most agents that prolong QT, TdP associated with amiodarone treatment has been shown to be higher in women than in men.

Cardiac glycosides are also potent inhibitors of HERG expression at the cell surface, reducing IKr currents, and prolonging repolarization and the QT interval. Digitoxin, ouabain, and digoxin appear to inhibit IKr by reducing the density of HERG-dependent potassium channels in the cell membrane. The reduced number of potassium channels in the cell membrane results from abnormal channel trafficking due to inhibition of channel exit from the endoplasmic reticulum [60] (Table **2**).

The protective effects of a rapid rhythm on TdP are well documented. Dofetilide a Class III antiarrhythmic induced more QT prolongation after rhythm conversion than during rapid-rate atrial fibrillation. Cardioversion of atrial fibrillation is known to increase the risk of long QT; whereas persistent atrial fibrillation has a protective effect for drug-induced long QT [5]. Additionally, slower heart rates are known to increase risk of TdP by QT prolonging drugs, some forms of congenital LQTS and hypokalemia. Similarly, autonomic block was shown to increase ibutilide-induced QT prolongation [61].

## USE OF QT-PROLONGING DRUGS FOR THE TREATMENT OF THE SHORT QT SYNDROME

Short QT syndrome is a new genetic disorder associated with a high rate of familial sudden death [62, 63]. Several mutations in genes encoding for cardiac ion channels (KCNH2, KCNQ1, and KCNJ2) have been identified to cause the short QT syndrome. These mutations lead to an increase in the IK currents (IKr, IKs, and IK1), which in turn shorten the action potential and the QT interval (<335 ms after correction for heart rate at rates <80 beats/min). Although an implantable cardioverter defibrillator is often the therapy of choice, pharmacological treatment with QT prolonging drugs has also been attempted. In a study of patients carrying a mutation in the IKr-coding gene KCNH2 (HERG), quinidine was found more effective than sotalol and ibutilide in prolonging the QT interval and normalizing the effective refractory periods [62]. The superiority of quinidine over Class IC and Class III drugs was confirmed in another study [63], where hydroquinidine was found to prolong the QT interval from 263 +/- 12 ms to 362 +/- 25 ms, and to prevent the likelihood of ventricular fibrillation. Therefore, drugs known to prolong the QT interval, quinidine in particular, can be effective in the treatment of short QT interval syndrome.

## NON-ANTIARRHYTHMIC DRUGS ASSOCIATED WITH INCREASED RISK OF TDP

Although for the class III antiarrhythmics prolonged cardiac repolarization is a desired effect, for other agents the blockade of repolarization is an unwanted side effect (Table **4**). The use non-antiarrhythmic QT-prolonging drugs increases by 2-fold the risk of cardiac arrest in hospitalized patients with several underlying diseases [64]. Such a risk was more pronounced in patients receiving more than one daily dose of the drug, more than one QT-prolonging drug, and drugs that interfere with the metabolism or elimination of the QT prolonging agent [64]. For QT prolonging orally administered drugs TdP developed within 3 days in only 18% of cases, between 3 and 30 days in 42% of cases, and after 30 days of treatment initiation in 40% of cases [65]. TdP associated with intravenous drug administration generally occurred at the time of peak plasma levels [65].

For many drugs, QT prolongation is described in the FDA-approved labeling as a known drug action, and for others, the FAD-approved labeling includes cases or a risk of TdP. Such information may be accessed at http://www. Torsades.org. [6] (Table **4**).

Antibiotics are among the non-antiarrhythmic therapeutic agents known to prolong the QT interval and to be associated with TdP. Macrolide antibiotics such as erythromycin, clarithromycin and azythromycin have reported to prolong the QT interval. However, greater risk of TdP has been observed erythromycin and clarithromycin, in particular at high doses, and during intravenous administration. Little to no risk to TdP has been reported with azythromycin despite QT prolongation. Differences in arrhythmogenesis may arise from differences in drug-induced changes in the morphology of the ventricular action potentials; with a more triangular morphology observed with erythromycin and clarythromycin, and a more rectangular morphology with azythromycin (see [67] for details) (Table **1**). In addition, to their direct effect on the QT interval erythromycin and clarithromycin exert inhibitory effects on drug metabolism by inhibiting Cyt P450 3A4. Macrolides should be avoided in patients taking pimozide or drugs known to prolong the QT interval (.e., class Ia, and III antiarrhythmics), and in subjects with underlying cardiac disease. High doses of macrolides should be avoided, and it has been recommended that serum K^+^ abnormalities be corrected prior to its use, particularly in patients with increased risk for TdP [66].

Antibiotics of the fluoroquinolone class may induce QT prolongation and increase the risk of arrhythmia [68-70]. The inhibitory potency of fluoroquinolones to inhibit HERG K^+^ channels was assessed *in vitro* employing patch-clamp electrophysiology techniques. All fluoroquinolone tested inhibited the channel, but with widely differing potencies. Sparfloxacin was the most potent compound, followed by grepafloxacin > moxifloxacin > gatifloxacin > levofloxacin > ciprofloxacin > ofloxacin. In the cases of levofloxacin, ciprofloxacin, and ofloxacin, inhibition of HERG was found to occur at concentrations much greater than those observed clinically. Sparfloxacin was not only the most potent in inhibiting Ikr, but in addition it increased the instability of repolarization [71]. Sparfloxacin, gatifluoxazine and moxifloxacin should be avoided in patients with risk factors for QT prolongation, and the recommended dose should not be exceeded. In general, use of any fluoroquinolone should be avoided in subjects with LQTS, subjects treated with class Ia and III antiarrhythmics or with any other agent known to prolong the QT interval [72]. Similarly, fluoroquinolones should be administered with caution and under ECG monitoring in cardiac disease (ischemic heart disease, CHF, bradycardia and rhythm disturbances) and serum K^+^ abnormalities should be corrected prior to administering fluoroquinolones [66, 68, 73].

Pentamidine, an antiprotozoal agent used in the treatment of trypanosomiasis, lehismaniasis and *Pneumocistis carinii* infections, is known to induce marked QT prolongation and arrhythmia [74]. Eisenhauer and colleagues [75] reported that in HIV patients with no apparent risk for QT prolongation a 1-hr infusion of pentamidine (4 mg/kg/d) prolonged the QTc interval by more than 120 msec in 5 of 14 subjects. Three of the five subjects developed TdP, one of whom died. Recent findings suggest that pentamidine-induced QT prolongation results from inhibition of channel trafficking and reduction in membrane channel density, rather than to direct channel blockade [76]. Geldalamicin, a benzoquinoid antibiotic, has also been shown to inhibit IKr currents by reducing trafficking of channels to the cell membrane [45].

Several other drugs have been reported to prolong the QT interval and to increase the risk of TdP. Cisapride, felbamate, forcarnet, tacrolimus, probucol, indapamide, moexipril and tizanidine, arsenic trioxide, methadone, octreotide, dolasetron and halofantrine, are among them [77]. Interestingly, the use of serotonin agonists of the tryptan class (sumatriptan, naratriptan and zolmitriptan) has also been associated with increased risk for TdP. Strong inhibition of IKr has been observed with cisapride and terfenadine. Among the antihistaminics, loratadine, terfena-dine and astemizole have been shown to block IKr; however, loratadine has little to no effect on the QT interval [78]. The later results from the 300-fold lesser potency of loratadine than of terfenadine and astemizole, in blocking the HERG K^+^ channels. Ranolazine has been shown to induce a modest increase of the QT interval, but apparently not associated with increase risk of TdP [79].

Domperidone, a dopamine (D2) receptor antagonist with prokinetic activity, has been shown to prolong the QT interval [80]. Its use was shown to increase the relative risk of sudden cardiac death of 3.8 fold [81], and the intravenous formulation was withdrawn from the market due to cardiac arrhythmias. Domperidone is metabolized by CYP3A4 and marked increases in its plasma levels, long QT intervals and arrhythmia have been reported when given combined with ketoconazole [82]. Interestingly, although structurally unrelated, most prokinetic agents (cisapride, domperidone and erythromycin) prolong the QT interval and its use is associated with arrhythmogenesis.

Drugs used for the treatment of psychosis also share arrhythmogenic potential related to repolarization abnormalities and QT prolongation. Sudden unexpected death has been reported to be nearly twice as frequent in populations treated with antipsychotics [83, 84]. Chlorpromazine, thioridazine, mesoridazine, risperidone, zisapridone, haloperidol, droperidol, sertindole, quetiapine and pimozide are some of the agents associated with prolongation of the QT interval. Of the phenothiazines tested, thioridazine was the most potent and chlorpromazine the least potent in blocking HERG K^+^ channels [85]. TdP associated with intravenous haloperidol administration is commonly observed within 15 to 220 min of drug administration [86]. Pimozide, sertindole, haloperidol and droperidol have been documented to cause TdP and sudden death. However, highest risk seems associated with thioridazine and droperidol use [79, 83]. Of the antipsychotics, butyrophenones and diphenylbutylpiperidines were reported more potent than the dibenzoxazepines as HERG K^+^-blocking agents; substituted benzamides exhibited the lowest blocking activity [87]. In addition to antipsychotics, drugs with antidepressant properties may also prolong the QT interval; among them: desipramine, imipramine, doxepin, fluvoxamine, fluoxetine, paroxetine, sertraline, doxepin and venlafaxine [87, 88]. Both fluoxetine and its metabolite, norfluoxetine, have been shown to directly inhibit the potassium channels and in addition, disrupt channel protein trafficking reducing the number of channels in the cell membrane [89] (Table **2**).

Arsenic trioxide has increasingly been used in the treatment of acute promyelocytic leukemia. Arsenic trioxide is commonly administered daily as a 2-hour infusion of 0.15 mg/kg in patients with relapsed or refractory acute promyelocytic leukemia. Such a treatment is known to prolong the QT interval, and is frequently associated with nonsustained ventricular tachycardia, requiring additional antiarrhythmic therapy [90]. Therefore, patients treated with arsenic trioxide should be monitored for prolonged QT intervals, presence of T-U wave alternats and ventricular arrhythmia [26]. Arsenic trioxide prolongs the QT interval by inhibiting the channel trafficking from the endoplasmic reticulum to the cell membrane. The compound seems not to directly block the potassium channels [91] (Table **2**).

Anthracyclines have been shown to be associated with TdP and to increase the sensitivity of IKr-blocking drugs to induce an arrhythmia; the later may be observed soon after initiation of anthracyline treatment. It has been proposed that anthracyclines reduce the repolarization reserve increasing the likelihood of developing life-threatening proarrhythmia [92].

In addition to drugs with significant effects to the QT interval, the azole group of antifungals, ketoconazole, itraconazole, fluconazole, miconazole, posaconazole and voriconazole has been reported to cause important interactions with agents known to prolong the QT [93]. The azoles *per se* have inhibitory effects on the IKr, but in addition, and in particular, ketoconazole, miconazole and itraconazole, are known to interfere with the metabolism of many other compounds by inhibiting cytochrome P450-3A4. Therefore, when combined with QT-prolonging drugs that are metabolized by this cytochrome system, large increases in plasma levels may occur. In fact, most of the deaths related to treatment with cisapride, astemizole, quinidine and terfenadine may have resulted from concomitant administration with azole compounds [90]. Thus, administration of two QT-prolonging drugs together with high plasma levels of a QT-prolonging drug are likely to increase the risk of TdP. Recent studies demonstrated that ketoconazole prolonged the QT not only through direct blockade of the IKr, but also by reducing the density (number of) of HERG potassium channels in the cell membrane. The channel trafficking from the endoplasmic reticulum to the cell membrane is inhibited by ketoconazole. Therefore, and similarly to fluoxetine and norfluoxetine, ketoconazole-induced LQTS may be achieved by a combination of two effects; namely, *via* a direct inhibition of the potassium channel and by disrupting HERG protein trafficking [94] (Table **2**).

Beta-adrenergic receptor stimulation induced either by increased sympathetic stimulation, albuterol, terbutaline, salmeterol, isoproterenol, ephedrine, pseudoephedrine, phenylephrine, or phenylpropanolamine, increases (upregulates) the number of L-type calcium channels in cardiac fibers, facilitating the development of early afterdepolarizations in the presence of long QT. Therefore, in the presence of an already long QT, and irrespective of its etiology, administration of these agents may trigger a ventricular arrhythmia [see additional drugs to avoid in patients with long QT, under www.torsades.org or www.Qtdrugs.org [6].

## RISK FACTORS AND PREDICTORS OF DRUG-INDUCED TDP

Women commonly have longer QTc intervals than males and have a greater propensity to develop TdP than men [15, 95, 96] (Table **1**). Nearly two-thirds of the cases of drug-induced TdP occur in women. Clinical and experimental studies show that female gender is associated with a longer corrected QT interval at baseline and a greater response to drugs that prolong the QT interval. Even in the presence of a drug that mildly blocks IKr and seldom or mildly prolongs the QT interval, women are still more prone to drug-induced TdP [5, 95]. Further, genetic defects of K^+^ channels that may be asymptomatic in normal conditions may precipitate drug-induced arrhythmia in women more frequently than in men. It has been proposed that women have a reduced cardiac 'repolarisation reserve' possibly due to an effect of estrogens. In fact, estrogens are known to facilitate bradycardia-induced prolongation of the QT interval and emergence of arrhythmia, whereas androgens shorten the QT interval and blunt the QT response to drugs [95].

Amiodarone, bepridil, quinidine, disopyramide, ibutilide, sotalol, erythromycin, pimozide, probucol, cisapride, terfenadine and halofantrin are among the drugs reported to increase risk of TdP in women [15, 95, 96].

Prolonged QT in the elderly is strongly associated with the development of ventricular arrhythmias [97]. As previously described, in addition to gender and older age, presence of congenital long-QT syndrome, bradycardia, systolic dysfunction, HIV, hypokalemia and hypomagnesemia are well-known risk factors for drug-induced TdP. Coexistence of several of these factors in an individual increases the risk of drug-induced TdP (Table **1**).

A previous history of drug-induced TdP predicts excessive QT prolongation when exposed to a QT-prolonging drug [52] (Table **1**). The QT prolonging effects of D, L-sotalol (2mg/kg, i.v.) were assessed in subjects with a history of TdP in association with QT-prolonging drug use and in age and sex matched controls. Although there were no differences in baseline QTc intervals between groups, sotalol increased QTc interval duration from 422+/-17 to 450+/-22ms in controls, and from 434+/-20 to 541+/-37ms in study subjects. TdP occurred in 3 of 20 in the study population but in none of the controls [52]. Assuming no differences in sotalol pharmacokinetics among groups, the findings suggest that subjects with a history of drug-induced TdP despite normal QTc at baseline have subclinical repolarization defects that were unmasked by sotalol. The findings also indicate that a normal QTc at baseline does not preclude excessive QT prolongation, nor development of TdP when exposed to a QT-prolonging drug [52]. Therefore, a previous history of drug-induced TdP, even in the presence of a normal QTc interval duration, should preclude further use of QT prolonging drugs. It is quite possible that subclinical ion channel mutations may account for the increased risk of QT lengthening and TdP observed in many individuals.

The presence of repolarization inhomogeneity assessed through measurements of QT dispersion has also been proposed as a predictor for drug-induced TdP. Chevalier and colleagues reported greater QT dispersion in subjects with a history of drug-induced transient QT lengthening >600 ms and/or TdP than in controls [98].

To determine whether relatives of subjects with a history of long QT are at increased risk for QT prolongation, intravenous quinidine was given to relatives of subjects who developed long QT after administration of QT-prolonging drugs and to relatives of subjects who failed to prolong the QTc interval (controls) [99]. No differences in QT between groups were observed at baseline or after quinidine. However, the interval from the peak to the end of the T wave, an index of transmural dispersion of repolarization, prolonged significantly with quinidine in first-degree relatives of patients who prolonged the QT interval, but not in control relatives. Although the study was conducted in a small number of subjects, the findings suggest the existence of a genetic predisposition to acquired (drug-induced) long QT [99] (Table **1**). To what extent the observed T wave abnormality indicates increased risk for developing TdP deserves further work.

## PRACTICAL ASPECTS

The following information should be collected prior to administer a QT-prolonging drug in order to prevent or minimize risk of drug-induced TdP:

1. Past Medical History. Rule out congenital long QT syndrome, history of arrhythmia and/or syncope either spontaneous or associated with drug use, presence of ischemic heart disease, cardiomyopathy and/or systolic dysfunction-heart failure, or HIV. Relatives of subjects with history of congential or acquired TdP or drug-induced long QT may also be at increased risk.
2. Medication history. Determine drug-use associated with previous arrhythmia/syncope or long QT. A previous history of drug-induced TdP should preclude the use of a QT prolonging drug. On current medication list, check for QT-prolonging drugs used alone or combined with inhibitors of drug-metabolism. Use of two or more drugs known to prolong the QT should be avoided. Beta-receptor agonists and sympathomimetics should also be avoided in subjects with increased risk.
3. Identify additional risk factors and combination of risk factors. Women are at greater risk of TdP than men. Elderly women on antipsychotic or anti-depressant medication, needing antiarrhythmic and/or antibiotic treatment must be carefully monitored. Avoid high doses of QT prolonging drugs.
4. Baseline ECG. Examine for heart rate (bradycardia), QTc duration and dispersion, presence of T wave abnormalities, U waves, and T wave alternants. QTc values greater than 450 msec for men and greater than 470 msec for females are usually considered as abnormally prolonged. Estimate QT dispersion using the difference between the maximum and the minimum QTc in any thoracic lead. Values greater than 80 msec are considered abnormally prolonged. When prolonged QT is associated with increased QT dispersion use of drugs that may further prolong the QT interval must be avoided, since such a combi-nation is known to increase the risk for developing drug-induced TdP [98].
5. Obtain serum K^+^, magnesium and creatinine levels before starting treatment with a QT-prolonging drug. Normalize serum K^+^ and magnesium if low. Assure normal renal function when administering QT prolonging drugs that are mainly eliminated by the kidney.
6. Treatment and follow-up plan. Avoid high doses of QT prolonging drugs and combinations of drugs known to prolong the QT interval. If a QT prolonging drug must be used, use it at low doses and avoid high peak levels and intravenous administration. Monitor QT on ECG during treatment. Avoid combined use of long QT drugs with drugs that inhibit their metabolism and may induce accumulation and high plasma levels.
7. Incorporate genetic testing in high-risk populations (family history, previous multiple syncopal episodes, previously documented TdP arrhythmia), as well as provocation tests (i.e, epinephrine QT test) [100]. Epinephrine QT stress testing is an effective diagnostic tool to unmask concealed long QT syndrome. Better understanding of HERG channel function and regulation is needed to diagnose and treat cardiac repolarization disorders and the development of severe and potentially fatal arrhythmias.

## Figures and Tables

**Table 1:** Factors Associated with Increased Risk of Developing Drug-Induced Excessive QT Prolongation and/or TdP

EEG changes [congenital or acquired]: Long QT interval [20-22, 33-35] Increased QT dispersion [12, 23-25, 98] Increased interval from peak to end of T wave [26,99] T wave alternans [26] T-U waves [26] Action Potential Prolonged [1-5] Prolonged with triangular shape [67] Electrolyte abnormalities Hypokalemia [46-48] Hypomagnesemia [101] Women [5,15,95,96] Older age [97] HIV [32] Systolic dysfunction [102] Previous history of drug induced long QT or TdP [52] Relatives of subjects with a history of drug-induced long QT [99]

**Table 2:** Mechanisms of Drug-Induced HERG-K^+^ Channel Inhibition, QT Interval Prolongation and Increased Risk for TdP

Drugs	Direct Inhibition of HERG K^+^ Channel	Inhibition of HERG K^+^ Channel Trafficking
Phenothiazines	+	?
Pentamidine	-	+
Geldalamicin	-	+
Arsenic Trioxide	-	+
Fluoxetine, Norfluoxetine	+	+
Ketoconazole	+	+
Digitoxin, Ouabain, Digoxin	-	+
Sparfluoxacin, Ciprofloxacin, Ofloxacin	+	-
Amsacrin	+	-
Probucol	-	+
Cisapride	+	

Drug-induced HERG-K^+^ channel inhibition can be achieved either by direct inhibition of potassium channels and/or by reducing the number of channels on the cell membrane. The later is achieved by inhibiting HERG-channel trafficking from the endoplasmic reticulum to the cell membrane. Data on drug-induced inhibition of channel trafficking is not available for all drugs. Therefore, the table depicts available data for agents that have been tested both for direct HERG-channel blockade and for interference with channel trafficking [Refs. 77,103-107].

**Table 3:** Antiarrhythmic Drugs Associated with QT Prolongation and Increased Risk for Developing TdP

Type III Agents	Other Anti-Arrhythmics With Type III Activity
Ibutilide	Quinidine
Dofetilide	Procainamide
Azimilide	Disopyramide
Bretylium	Propafenone
Amiodarone	Bepridil
D-sotalol	Prenylamine
Tedisamil	Flecainide
	Terodiline Digoxin

**Table 4:** Some Non-Antiarrhythmic Drugs that have been Reported to either Prolong the QT Interval and/or to Increase the Risk for Developing TdP

Drug Class	Chemical Class	Drugs
Antibiotics	Macrolides	Eryhthomycin, Claritromycin, Telithromycin
	Fluoroquinolones	Sparfloxacin, Gemifluxacine, Grepafloxacin, Moxifloxacin, Gatifloxacin, Levofloxacin
	Benzoquinoid	Geldamicin
Antiprotozoals		Pentamidine, Chloroquine,Halofantrine
Antifungals		Ketoconazole, Itraconazole, Fluconazole, Miconazole, Posaconazole, Voriconazole
Antineoplastic		Arsenic trioxide, Anthracyclines, Sunitinib
Prokinetics		Cisapride, Domperidone, Erythromycin,
5HT3-recptor antagonists		Dolasetron, Granisetron, Ondansetron
Antihistaminics		Terfenadine, Astemizole
5-HT1D agonists		Sumatriptan, Naratriptan, Zolmitriptan
Antipsychotics	Phenothiazines	Thioridazine, Mesoridazine, Chlorpromazine
	Butyrophenones	Haloperidol, Droperidol
	Atypical Neuroleptics	Sertindole, Pimozide, Clozapine, Quetiapine, Risperidone
Antidepressants	Tricyclic antidepressants	Desipramine, Imipramine, Doxepin.
	Selective Serotonin Uptake Inhibitors	Paroxetine, Sertraline, Doxepin, Venlafaxine, Fluoxetine, Norfluoxetine, Fluvoxamine
Opiods		Levomethadyl, Methadone.
Drugs with cardiac and vascular effects		Ranolazine, Alfuzosin, Indapamide, Isradipine, Moexipril/HCTZ, Vardenafil
